# Every move counts: but some more than others

**DOI:** 10.1186/s12966-025-01865-x

**Published:** 2026-01-03

**Authors:** Melvyn Hillsdon, Brad Metcalf, John N. Newton, Amal A. Wanigatunga, Jennifer A. Schrack

**Affiliations:** 1 Department of Public Health and Sport Sciences, University of Exeter, St Luke’s Campus, Heavitree Road, Exeter, EX1 2LU UK; 2 European Centre for Environment and Human Health, University of Exeter, Peter Lanyon Building, Penryn, TR10 8RD UK; 3 Department of Epidemiology, Johns Hopkins Bloomberg School of Public Health, Baltimore, MD USA; 4 Center on Aging and Health, Johns Hopkins University, Baltimore, MD USA

**Keywords:** Physical activity, Time series data, Accelerometers, Health outcomes, Public health guidelines

## Abstract

**Background:**

Physical activity (PA) plays an essential role in reducing the risk of non-communicable diseases and improving health and well-being. However, current PA guidelines do not adequately reflect emerging evidence on the importance of the relationship between patterns of PA bout duration and health outcomes.

**Main text:**

This paper explores the limitations of evidence based on self-reported, aggregated measures of PA, and advocates for greater use of time series data from accelerometers to describe daily patterns of PA bout duration. Time series data offer insights into how PA is accumulated in different bout lengths and how such patterns impact health, independent of total weekly volume of PA. Evidence from accelerometer-based studies of the association between patterns of PA bout duration and health outcomes challenges the revised World Health Organisation guideline that ‘every move counts toward better health’.

**Conclusion:**

By highlighting the novel nature of time series data and their corresponding patterns of PA bout duration, this paper aims to challenge current public health guidelines and inform the development of future guidelines, surveillance, policies, and interventions to prevent morbidity and mortality.

## Background

The benefits of a physically active lifestyle are well established. Higher levels of physical activity (PA) are associated with a reduced risk of mortality, cardiovascular disease, diabetes, certain cancers, hypertension, obesity, improved mental well-being, and quality of life [1]. Despite this, a significant portion of the global population fails to meet recommended PA levels, contributing to substantial economic burdens in terms of healthcare costs and lost productivity [2]. Current PA guidelines advise adults to engage in at least 150–300 min per week of moderate intensity PA or 75 min of vigorous intensity PA, or a combination thereof, in bouts of any duration [3].

The evidence underpinning these guidelines comes primarily from studies that rely on self-report measures of daily or weekly physical activity engagement. These studies are challenged as they: (1) likely underestimate the true association between PA and health because of misclassification due to recall error or social desirability bias [4], and (2) tend to use aggregate or summary statistics such as time spent in moderate-to-vigorous intensity PA (MVPA), with little information on how PA is accumulated over time [5]. In 2018, the U.S. Physical Activity Guidelines removed the previously required minimum bout duration of 10 min, stating that “bouts, or episodes, of moderate-to-vigorous physical activity (MVPA) of any duration may be included in the daily accumulated total volume of PA [6].” Similarly, the World Health Organisation also removed the previously mandated minimum 10-minute bout duration requirement and adopted the principle that “every move counts towards better health [7].” This major change in public health guidance on PA was the first to be informed by studies utilizing body-worn accelerometers for estimating PA [6]. However, this interpretation reflects only one side of a broader discussion. An alternative perspective contends that more sustained, less fragmented bouts of PA are critical determinants of health and functioning, often beyond total PA [8]. This paper examines the contrasting arguments for and against the importance of bout duration, with the aim of clarifying their conceptual foundations, evidential support, and implications for future public health policy and guidelines.

## Main text

### Problems with self-report and aggregate measures

Recall error and social desirability bias are common in self-report measures and likely lead to misclassification in estimated levels of PA by population sub-group. This in turn leads to over- or underestimation of the differences between sub-groups depending on context. For example, people tend to better recall structured, organised PA (e.g., going to the gym, playing sport, going for a run etc.), compared to more routine daily activities accumulated in multiple short bouts throughout the day [9]. Structured sport and recreational PA are more common among younger, more affluent males, whereas everyday activities are more common among less affluent and older adults [10]. Accordingly, the resulting differential misclassification of PA could lead to false conclusions about PA inequalities and misdirected polices and resources.

In aetiological studies, commonly used daily aggregative measures are not designed to capture variations in patterns of PA accumulation, potentially masking associations between accumulation patterns and specific diseases or individual health trajectories. Aggregate measures can also lead to an underestimate of the effectiveness of interventions. If change in average steps per day is a primary outcome and no change in total steps per day is recorded at follow-up, it could be concluded that the intervention was ineffective. However, if participants in the intervention group increased their time in longer, more sustained (less fragmented) bouts of activity, this could lead to improvements in fitness, function and health, rendering the intervention effective.

Reliance on aggregate measures of daily PA also ignores potentially important differences in patterns of daily PA by sociodemographic groups. Sex, body mass index and type of employment (more or less physically active) have been associated with different patterns of PA accumulation independent of total volume of activity [11]. These differences in patterns of accumulation suggest that people in lower socioeconomic or age groups engage in less PA overall, but also accumulate their activity through more frequent, shorter bouts, which are not well captured by self-report measures. These constraints posed by self-report measures, coupled with the dependency on daily summaries of PA, present a challenge in accurately capturing such nuanced elements introduced in the updated PA guidelines, which encompass all movement, regardless of duration, as well as extended periods of sedentary behaviour. Consequently, most existing PA surveillance systems do not adequately capture everyday movements and patterns of sedentary behaviour.

### Use of accelerometers in physical activity measurement

Accelerometers offer a way to overcome many of the limitations associated with self-report measures. These small, portable, passive, non-invasive devices are low burden for participants and capable of collecting high resolution data remotely in real time. However, they are not without limitations [12]. Early accelerometers were used to measure vibrations in industry and in aeronautics in the 1920 s long before their use for the measurement of human movement in the 1970 s [13]. By the 1990 s they had become popular in epidemiological studies [14]. For example, they were used in the National Health and Nutrition Examination Survey (NHANES) between 2003 and 4 from which estimates of PA prevalence in the US were reported for the first time based on accelerometer data [15].

The use of accelerometers is now ubiquitous in epidemiological and intervention studies of PA, but less so in population surveillance studies where the numbers of subjects are much larger. Only a small number of countries have incorporated accelerometer measures into their existing PA surveillance systems [16].

Most derived measures from accelerometer data are designed to capture compliance with current public health guidelines. In the past, these advised at least 30-minutes of MVPA on at least 5-days of the week, so the accelerometer data was used to estimate the average number of minutes of MVPA per day [17]. This approach although understandable represents a massive underutilisation of the high resolution, minute-by-minute, even second-by-second data potentially available. The use of derived aggregate measures may also mean that important associations between patterns of PA bout duration and health outcomes may be overlooked.

Time stamped movements are recorded by most accelerometers, multiple times per second, which can produce detailed time-series of contiguous active and inactive events. Accelerometer data therefore provides the opportunity to go well beyond aggregate measures to consider how daily PA is accumulated in bouts of different durations, as well as how much PA is undertaken. Although numerous metrics exist to characterise the ways in which PA is accumulated [18, 19], few have generated sufficient empirical evidence to inform policy, and many do not focus on bout duration. This discussion therefore, focuses on the two approaches supported by substantial evidence that offer contrasting interpretations of the importance of bout duration for health benefit.

**Fig. 1:**
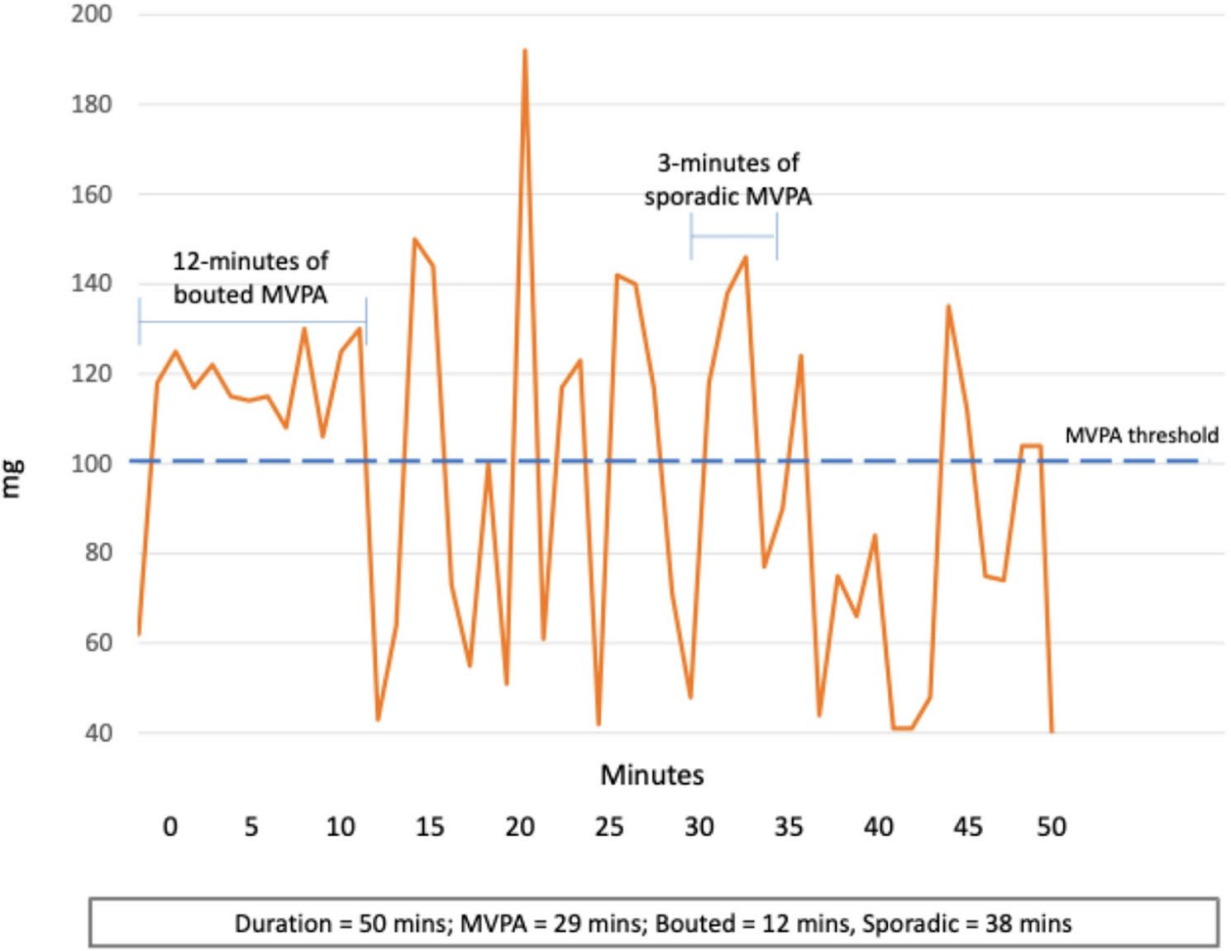
Illustration of the threshold-based approach to quantifying bout duration in moderate-to-vigorous physical activity (MVPA). Accelerometer data are summarised in 60-second epochs and classified as MVPA only when exceeding a defined intensity threshold (dashed line). Periods of continuous MVPA lasting ≥ 10 min are identified as bouted MVPA, while shorter, intermittent periods above the threshold are considered sporadic MVPA. In this example, total wear time is 50 min, with 29 min above the MVPA threshold—12 min classified as bouted MVPA and 17 min as sporadic MVPA. In contrast, the Active-to-Sedentary Transition Probability (ASTP) approach would treat the entire 50-minute sequence as a single sustained period of activity

**Fig. 2:**
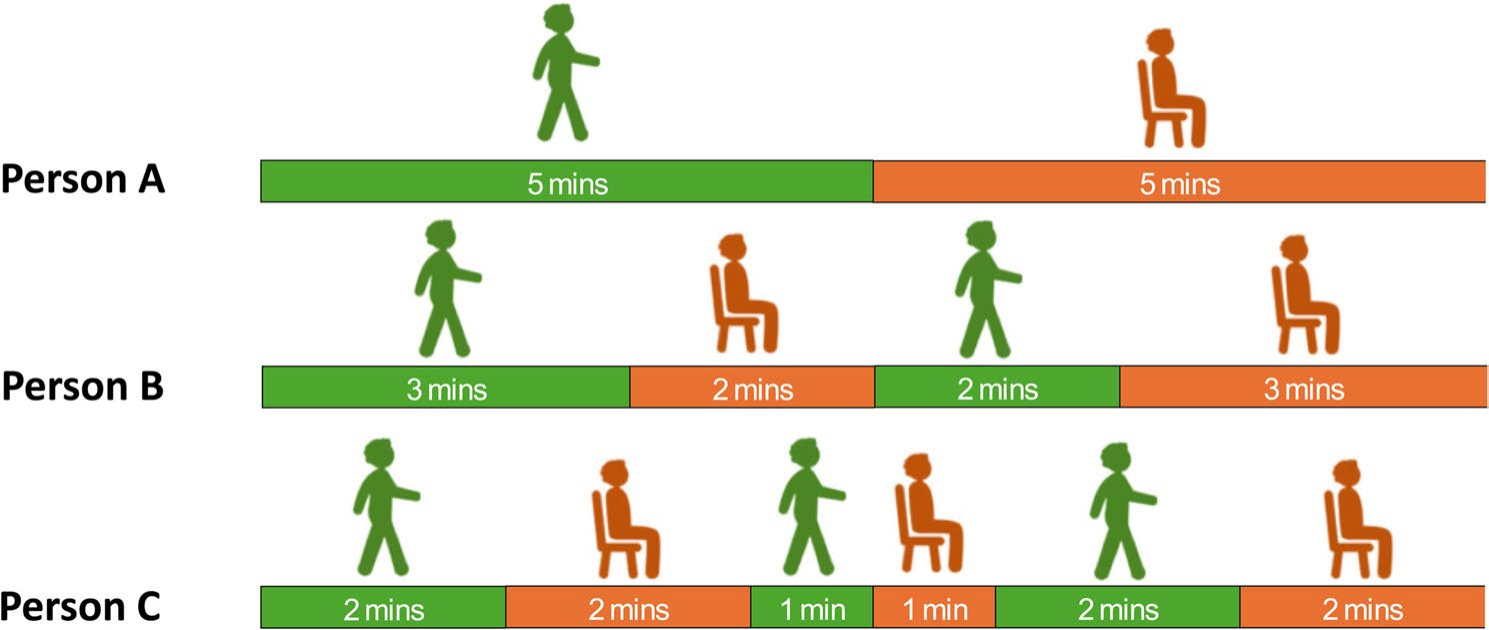
The same volume of PA accumulated in increasingly fragmented way. Figure 2 (Adapted from Wanigatunga AA, 2022) [22] illustrates three people all recording 5-minutes of activity and 5-minutes of inactivity, but accumulating the activity at different levels of fragmentation. Person A accumulates their walking in a single sustained bout with an ASTP of 0.2 (a 20% probability that an active minute will be followed by a period of inactivity), whereas Person B accumulates their walking in two bouts with a rest in between and an ASTP of 0.4. Person C has 3 transient bouts, interspersed with periods of rest with an ASTP of 0.6

To guide readers through the methodological and conceptual distinctions central to this discussion, Tables 1 and 2 and Figs. 1 and 2 summarise the main approaches used to characterise the structure of PA bouts—that is, the mix of shorter and longer periods of movement that together form an individual’s activity pattern. Table 1/Fig. 1 outline the conventional, threshold-based method used to define ‘*bouted’* (sustained*)* and *‘sporadic’ (fragmented)* MVPA [20, 21], while Table 2/Fig. 2 illustrate the more recent transition-based approach represented by the Active-to-Sedentary Transition Probability (ASTP)[22]. Both methods utilise time series of 60-second epoch data derived from the raw acceleration. While more advanced time series methods have been used [18, 23], at present, these two methods are the most common and easiest to compute. Together, these frameworks highlight two complementary ways of quantifying how sustained or fragmented bouts of PA are, with each addressing the central question of this discussion: does the duration of activity bouts matter for health, and should more sustained bouts of activity be included in public health guidance?

**Table 1:** How conventional threshold-based bouts of MVPA are calculated

● Epochs of accelerometer data (commonly 60 seconds) are classified as MVPA when intensity exceeds a defined threshold (e.g., 100 mg) [24].
● BoutedMVPA is defined as sustained periods of consecutive MVPA epochs when a minimum duration is achieved (e.g., ≥10 minutes) and interruptions are shorter than a tolerance window (usually 20%, so when MVPA bout = 10 minutes no more than 2 of epochs are < 100 mg) [25].
● Total bouted MVPA is the sum of all MVPA minutes contained in such bouts; *sporadic* MVPA includes all remaining MVPA not part of a qualifying bout

**Table 2:** How Activity to Sedentary Transition Probability is calculated

● Epochs of accelerometer data (commonly 60-seconds**)**, are classified as active or sedentary according to established intensity thresholds (e.g., ≥40 mg) [25].
● The ASTP is calculated as:
$$ASTP=\frac1{N_{active\rightarrow sedentary\;transitions}/N_{active\;epochs}}$$
⚬ ASTP = 0 indicates no transitions from active to sedentary behaviour during the observation period – a single, continuous bout of activity.
⚬ ASTP = 1 indicates that every active minute is immediately followed by a sedentary minute – a completely fragmented pattern of activity.
● Lower ASTP values reflect a lower fragmented of bouts (more longer bouts and less shorter bouts of PA),
● Higher ASTP values reflect a greater fragmentation of bouts (more longer bouts and less shorter bouts of PA).

### Does bout duration matter?

The research underpinning the US and WHO guidelines were based on the methods presented in Table 1 and Fig. 1 and has led to the conclusion that bout duration does not matter [20]. However, this method has notable limitations. Individuals engaging in prolonged PA just below the MVPA threshold may be misclassified as performing sporadic MVPA (more fragmented). Similarly, individuals who engage in short bursts of high-intensity activity interspersed with lower-intensity movement during a prolonged bout may have their activity incorrectly labelled as sporadic (more fragmented), despite maintaining continuous movement (Fig. 1). The ASTP method, in contrast, categorizes these individuals as having low fragmentation. Further, individuals who accumulate their activity in these two ways (longer, lower intensity bouts or shorter high intensity bouts) may accumulate a greater total volume of activity than those classified as engaging in more bouted MVPA, which may be one reason why bout duration appears less important. Indeed, it has been estimated that 85% of the data labelled as sporadic MVPA is embedded in longer bouts of activity of mixed intensities [26].

Moreover, the conventional MVPA classification system allows for up to 20% of an MVPA bout to fall below the acceleration threshold, meaning that brief spikes of high-intensity activity separated by short periods of inactivity could be misclassified as sustained MVPA. This exaggerates the recorded duration of sustained activity that would be categorised as more fragmented by the ASTP method [27].

The decision to remove a minimum bout duration from national and international guidelines and state that “every move counts,” did not recognize that patterns of PA bout duration may be associated with health outcomes independent of total daily PA. Specifically, a measure of how often people shift between active and sedentary behaviours (e.g., PA fragmentation) has been consistently associated with health outcomes, often over and above total daily PA [8]. Specifically, in a cohort study of 548 older adults, with 4.4 years of follow-up, total daily PA was not associated with mortality risk, whereas a higher frequency of short bouts of activity (more fragmented PA) was associated with an elevated risk of mortality compared to longer, more sustained bouts (less fragmented PA) [28]. More fragmented PA was also associated with an increased mortality risk in a cohort study of 11,992 adults (18–74 years) followed for 11 years [29].

A given total volume of activity accumulated in more frequent, short bouts (fragmented PA) compared to less frequent, sustained bouts is associated with a range of less favourable health outcomes including poorer physical function, fatigability, cognitive impairment, frailty and mortality [30–33].

These findings underscore the importance of bout duration in determining impact on health outcomes, contrasting with the alterations made to national and international PA guidelines. The alterations suggest that fragmented MVPA confers similar health benefits to sustained MVPA, which appears contradictory to emerging evidence. Although we agree that all activity is beneficial to some extent, the duration of bouts of activity does matter, and this should be included in public health guidance.

In addition to PA fragmentation, other measures of patterns of PA accumulation have been associated with health. The period of day in which PA is accumulated is emerging as a factor that may influence mortality risk and some diseases. For example, a cohort study of 92,139 UK Biobank participants found that MVPA accumulated in the afternoon or uniformly across the day was associated with a lower risk of all-cause mortality compared to MVPA mostly accumulated in the morning [34], which is important because older adults tend to compress much of their PA into the morning period [5]. However, this is complicated by the fact that chronotype is associated with the timing of physical activity and many of the physiological mechanisms relating to physical performance [35–37]. Further, in a review of people with type 2 diabetes, there was evidence that PA undertaken soon after a meal was more beneficial for glucose control than PA taken at other times [38], likely by attenuating the peak and duration of the postprandial glucose excursion [39].

### Patterns of accumulation of PA as digital biomarkers

Changes in pattern of PA bout duration may also be useful as an outcome measure. A metric that reflects PA fragmentation, could be used as a digital biomarker for detecting pre-clinical signs of disease or accelerated ageing earlier in the pathological course [8].

For example, PA fragmentation may serve as a compensatory strategy to conserve energy amid functional decline and heightened fatigue commonly associated with ageing or disease progression. This fragmented activity pattern may also be a more sensitive indicator of decline compared to total PA or MVPA, as reductions in overall activity are likely to occur later, once compensatory mechanisms can no longer preserve energy or alleviate fatigue [40]. Changes in daily patterns of habitual PA are likely to start in mid-life among well-functioning adults and before any laboratory or clinic-based measures of physical function or disease would detect any change [8]. Unlike laboratory measures, measures of changes in patterns of PA accumulation can be undertaken remotely, at scale and at a time in the lifecourse when interventions could be effective at preventing or delaying progression to frailty and chronic disease. Importantly, what constitutes a clinically meaningful rate of change in fragmentation over time has not yet been established.

### Implications for public health

Most current surveillance systems for PA rely on self-reported measures and struggle to capture adherence to the revised aspects of the most recent public health guidelines. This necessitates measuring all bouts of PA regardless of duration, as well as monitoring the frequency and duration of sedentary behaviours. This level of detailed assessment is best achieved through minute-by-minute or even second-by-second data collection using research grade accelerometers. Although it is attractive to consider using commercial wearable devices, various limitations, including proprietary algorithms and lack of access to raw data [12], make them unsuitable for large-scale research. A complete transition to accelerometer-based measures for surveillance is not without challenges, including loss of historical trend data if existing systems are replaced [12, 41]. Recognising that no single method can fully capture the nuances of the new guidelines, a combined approach utilizing both self-report and device-based measures is recommended. This blended approach offers a more comprehensive understanding of how individuals integrate movement into their daily lives and enables a more precise assessment of inequalities in PA. Moreover, by incorporating both methods, surveillance systems can adapt to future guideline changes informed by evidence from device-based measures. Having said this, how best to integrate the two systems has yet to be determined.

Evidence from accelerometers using methods that allow more of the raw data to contribute to the analysis challenges some of the messages in current guidance. It underscores the importance of supplementing short, intermittent bouts of PA, which make up most daily activities, with more sustained bouts. We are not proposing a return to the previous 10-minute, minimum bout duration, but rather that the “every move counts” principle should be supplemented with at least one daily bout of longer, more sustained activity, such as an extended walk at a comfortable pace (akin to the daily mile schools’ program, https://thedailymile.co.uk) [42]. Some caution is required as it is possible that the promotion of longer bouts could inadvertently widen inequities if environments, living circumstances, or health restrict opportunities for engaging in longer bouts.

To date, whether changes in fragmented PA are a precursor to changes in endurance capacity, physical function etc., a compensatory mechanism, or both is unclear and highlights one of the limitations of the mostly observational data cited. Further studies across age ranges, with robust designs for dealing with confounding and causality are required. In addition, the metrics derived from the methods described can be affected by several accelerometer processing choices, and future harmonisation of processing pipelines would strengthen the evidence base and assist with translation to surveillance [43].

The potential benefits of integrating this knowledge into guidance are considerable. By promoting purposeful and sustained PA, particularly in mid-life, the onset of frailty and chronic disease could be delayed, leading to significant healthcare cost savings and allowing individuals to maintain their desired independence and quality of life for longer periods.

## Conclusion

Measuring daily PA in terms of amount and patterns of accumulation will lead to a deeper understanding of the role PA plays in keeping people healthy, providing more meaningful estimates of population-level PA, and informing more targeted public health messages and interventions. Harnessing the power of time series data from accelerometers marks a paradigm shift away from simple summary measures of PA volume toward a more detailed approach to capture the diverse ways people incorporate movement into their daily lives. Early changes to how we move (more fragmented) - occurring before changes in *how much* we move - may be a prognostic indicator that our ability to do the things that we value in life is declining. Finding the earliest possible signs of such premature loss of functional capacity is critical to reducing pressure on overburdened health and care systems, as well as keeping people socially engaged and economically active for longer in the lifecourse.

## Data Availability

No datasets were generated or analysed during the current study.

## Abbreviations

PA: Physical activity
MVPA: Moderate to vigorous physical activity
NHANES: National Health and Nutrition Examination Survey
ASTP: Active-to-Sedentary Transition Probability
